# Associations of Linear Growth and Relative Weight Gain in Early Life with Human Capital at 30 Years of Age

**DOI:** 10.1016/j.jpeds.2016.12.020

**Published:** 2017-03

**Authors:** Bernardo Lessa Horta, Cesar G. Victora, Christian Loret de Mola, Luciana Quevedo, Ricardo Tavares Pinheiro, Denise P. Gigante, Janaina Vieira dos Santos Motta, Fernando C. Barros

**Affiliations:** 1 Postgraduate Programme in Epidemiology, Universidade Federal de Pelotas, Brazil; 2 Postgraduate Program in Health and Behavior, Universidade Católica de Pelotas, Brazil

**Keywords:** intrauterine growth, childhood growth, intelligence, schooling, income

## Abstract

**Objective:**

To assess the associations of birthweight, nutritional status and growth in childhood with IQ, years of schooling, and monthly income at 30 years of age.

**Study design:**

In 1982, the 5 maternity hospitals in Pelotas, Brazil, were visited daily and 5914 live births were identified. At 30 years of age, 3701 subjects were interviewed. IQ, years of schooling, and income were measured.

**Results:**

On average, their IQ was 98 points, they had 11.4 years of schooling, and the mean income was 1593 reais. After controlling for several confounders, birthweight and attained weight and length/height for age at 2 and 4 years of age were associated positively with IQ, years of years of schooling, and income, except for the association between length at 2 years of age and income. Conditional growth analyses were used to disentangle linear growth from relative weight gain. Conditional length at 2 years of age ≥1 SD score above the expected value, compared with ≥1 SD below the expected, was associated with an increase in IQ (4.28 points; 95% CI, 2.66-5.90), years of schooling (1.58 years; 95% CI, 1.08-2.08), and monthly income (303 Brazilian reais; 95% CI, 44-563). Relative weight gain, above what would be expected from linear growth, was not associated with the outcomes.

**Conclusion:**

In a middle-income setting, promotion of linear growth in the first 1000 days of life is likely to increase adult IQ, years of schooling, and income. Weight gain in excess of what is expected from linear growth does not seem to improve human capital.

In low- and middle-income countries, rapid weight gain in early childhood has clear short-term benefits, including the reduction of morbidity and mortality owing to infectious diseases.^1^ In contrast, the evidence on the long-term consequences of rapid weight gain in childhood are not as clearcut. Several studies, mostly from high-income countries, reported that rapid weight gain in childhood is associated with a greater risk of obesity^2^, ^3^, ^4^, ^5^ and hypertriglyceridemic waist phenotype.^6^

The associations of weight gain in childhood and metabolic cardiovascular risk factors in low- and middle-income countries seem to be different. In a pooled analysis of data from 5 birth cohorts, weight gain in the first 2 years of life was associated with higher fat-free mass in adulthood, whereas weight gain after the first 2 years of life was associated with higher fat and fat-free mass.^7^ Norris et al^8^ used data from the same 5 cohorts and found that impaired fasting glucose and type 2 diabetes were not associated with weight gain in the first 4 years of life, whereas weight gain from 48 months increased the risk. These and other studies^9^, ^10^, ^11^ stressed that the long-term consequences of weight gain in childhood depend on timing, with negative consequences being particularly associated with late weight gain rather than gain in the first 1000 days.

With respect to human capital, different indicators of malnutrition (wasting, underweight, and stunting) in early childhood are associated negatively with performance on cognitive tests and years of schooling completed later in childhood and adolescence.^12^, ^13^, ^14^, ^15^, ^16^, ^17^ Furthermore, children who recovered from stunting performed slightly better on cognition tests than those who remained stunted, but less well than those who did not experience stunting.^18^, ^19^ Additionally, Hoddinott et al^20^ reported that the height-for-age *z*-score at 2 years of age was positively associated with years of schooling, performance on cognitive testing, and socioeconomic status in adulthood.

With respect to growth in childhood, school achievement and IQ are associated more strongly with early than with later growth in childhood.^21^, ^22^, ^23^, ^24^, ^25^, ^26^ In contrast, Krishnaveni et al^27^ did not observe an association between linear growth in childhood and early adolescence and IQ in adolescence. Most of these studies relied on weight gain as the exposure variable, and until recently there was no attempt to disentangle the consequences of weight gain from those of linear growth. It is important to disentangle the effect of weight from that of linear growth because they may have different consequences. In 2013, in a pooled analysis of 5 cohort studies from low- and middle-income countries (including data from the 23-year follow-up visit to the 1982 Pelotas cohort), Adair et al^9^ reported that linear growth in the first 2 years of life was associated more strongly with years of schooling than weight gain, suggesting therefore that nutritional interventions in childhood should be focused in promoting linear growth instead of weight gain. Moreover, growth monitoring programs should also incorporate length/height measurements.

The present study aimed to evaluate how birthweight, nutritional status, linear growth, and relative weight gain in childhood are associated with performance in intelligence tests, years of schooling, and monthly income at 30 years of age.

## Methods

In 1982, the maternity hospitals in Pelotas, a southern Brazilian city, were visited daily and all in-hospital births were identified. The 5914 liveborns whose families lived in the urban area of city were examined and their mothers interviewed. In 1984 and 1986, all households located in urban areas of the city were visited in search of cohort members; 5161 and 4979 individuals were evaluated in 1984 and 1986, respectively. From June 2012 to February 2013, cohort members were invited to visit the research clinic to be interviewed and examined. We interviewed 3701 subjects, which added to the 325 known to have died, for a follow-up rate of 68.1%. Concerning the losses to follow-up, we were unable to locate 1055 participants from the original cohort, 467 were living far from Pelotas, 86 refused to take part in this follow-up, and 280 did not attend the clinic despite repeated invitations. Further details on the study methodology have been published elsewhere.^28^, ^29^ The Ethical Review Board of the Faculty of Medicine of the Federal University of Pelotas approved the study, and written informed consent was obtained from all participants.

Birthweight was assessed by the hospital staff using pediatric scales that were calibrated weekly by the research team. Gestational age estimate was based on the mother's recall of the date of her last menstrual period. Preterm birth was defined by a gestational age of <37 weeks. In the 1984 and 1986 visits, children were weighed using calibrated scales and their length and height were assessed with portable stadiometers. Weight and height *z*-scores, according to age and sex, were estimated using the 2006 World Health Organization growth standards.^30^ Birthweight for gestational age *z*-scores were calculated using the Williams reference population.^31^

### Outcomes

Performance in intelligence tests was evaluated in the 2012-2013 visit, at a mean of 30.2 years of age. Four psychologists who were unaware of participant intrauterine growth and nutritional status in childhood administered the Wechsler Adult Intelligence Scale, third version, which has been validated for the Brazilian population.^32^ The following subtests were used: arithmetic, digit symbol, similarities, and picture completion.

Subjects were asked about the highest grade completed successfully at school, as well as their income in the previous month in Brazilian reais.

### Conditional Growth

Conditional growth modeling was used in the analyses on the effect of weight and height gain.^33^ Conditional variables were obtained by regressing current size (weight or length/height) on birthweight and earlier measures of weight and length/height, and standardized residuals were derived. Conditional variables express how a child deviates from its expected height or weight, based on its previous measures and the growth of the studied population. At each time point, the conditional variable represents growth during a time interval, and a positive value represents a weight gain or linear growth faster than predicted in that period. For example, conditional relative weight gain at 2 years of age represents the relative weight from birth to 2 years of age. The conditional variable at 4 years of age represents height or relative weight gain from 2 to 4 years of age. To estimate conditional height, current length or height was regressed on previous weight and length. Therefore, conditional length at 2 years of age was estimated by regressing length-for-age *z*-scores at 2 years of age on birthweight. In contrast, conditional relative weight was estimated from length/height at that age and previous measures of length/height and weight. Therefore, conditional relative weight at 2 years of age was derived by regressing weight at 2 years of age on birthweight and length at 2 years of age.

### Confounding Variables

Family income at birth was defined as total income earned by the family members in the month before the interview. Maternal and paternal years of schooling at birth was defined as years of schooling successfully completed. Household assets index in childhood was defined as based on the ownership of household goods and estimated using factor analysis.^34^ Maternal skin color was rated by the interviewer during the perinatal study. Maternal smoking during pregnancy was defined as those mothers with a history of smoking in the pregnancy being considered smokers. Breastfeeding duration was based on information on breastfeeding duration was collected in 1984 and 1986, and we used the information closest to the age of weaning to minimize recall bias.

### Data Analyses

ANOVA was used to compare means and multiple linear regression to obtain estimates that were adjusted for the following confounders: family income at birth, maternal years of schooling at birth, paternal years of schooling in childhood, household assets index, maternal skin color, and maternal smoking during pregnancy. Estimates on the effect of nutritional status in childhood and conditional growth were further adjusted to breastfeeding duration. Furthermore, for conditional length gain from birth to 2 years of age, estimates were also adjusted for birthweight according to gestational age *z*-score, whereas for conditional relative weight gain from birth to 2 years of age, analyses also controlled for conditional length gain from 0 to 2 years of age, for conditional length gain from 2 to 4 years of age, birthweight and conditional variables from 0 to 2 years of age were also controlled for, and for relative weight gain from 2 to 4 years of age, length gain from 2 to 4 years of age was also included in the model. Statistical comparisons between groups were based on tests of heterogeneity and linear trend in the case of ordinal variables, and the one with the lower *P* value was presented. The suest command was used to compare regression coefficients. We tested for interaction between birthweight for gestational age *z*-scores and conditional growth variables.

## Results

In the 2012-2013 visit, 3701 subjects were interviewed; added to the 325 known to have died, this represents a follow-up rate of 68.1%. Information on IQ at 30 years of age was available for 3611 subjects (61.1% of the original cohort), whereas complete data on IQ and nutritional status at birth and 2 and 4 years of age, were available for 2477 individuals (41.9% of the original cohort). Subjects with complete data were more likely to be female, to have been born in the intermediate socioeconomic groups, and have a birthweight of ≥2500 grams (Table I; available at www.jpeds.com). The mean IQ of those subjects with full growth data was 98.9 points (95% CI, 98.4-99.4), compared with 96.0 (95% CI, 95.2-96.7) among those who did not have full anthropometric assessments.

About 1 of every 3 mothers had ≤4 years of schooling, and most of the sample belonged to low-income families at birth (1 minimum wage in 1982 corresponded with US$50.00). The prevalence of low birthweight (birthweight < 2500 grams) was 7.2% and 14.2% of the studied subjects had a birthweight that was <10th percentile according to gestational age and sex. Concerning nutritional status in childhood, 12.8% of the subjects had a height-for-age *z*-score of ≤−2 SD at 2 years of age. The mean IQ at 30 years of age was 98 points and the average number of years of schooling was 11.4 (Table II; available at www.jpeds.com).

With respect to the confounding variables, socioeconomic status was associated inversely with the prevalence of low birthweight, small-for-gestational age, and low weight and height for age *z*-score at 2 and 4 years of age. The proportion of children whose conditional length at 2 years of age variable was ≥1 *z*-score was positively associated with socioeconomic status, whereas conditional height at 4 years of age and conditional weight variables were independent of socioeconomic variables (Table III; available at www.jpeds.com). IQ, years of schooling, and income at 30 years of age were positively associated with socioeconomic status at birth and childhood. Maternal smoking during the pregnancy was associated with lower IQ, years of schooling, and income at 30 years of age (Table IV; available at www.jpeds.com).

In unadjusted analyses, birthweight was positively associated with IQ, years of schooling, and income at 30 years of age. Controlling for confounding variables reduced the strength of the association, but subjects whose birthweight was ≥3500 grams still presented an higher IQ (2.93 points; 95% CI, 1.47-4.39) and income at 30 years of age (R$ 315; 95% CI, 91-538) than low birthweight subjects. The crude and adjusted results also show significant linear trends for years of schooling and income. Birthweight for gestational age was also positively associated with IQ and years of schooling, but not with income. In contrast, preterm birth was not associated with the outcomes (Table V).

Table VI shows that attained weight and length/height for age *z*-scores at 2 and 4 years of age were associated positively with the 3 outcomes. With the exception of the association between length for age at 2 years of age and family income in the adjusted model, all other associations were significant. Adjustment for confounding attenuated the magnitude of the associations, and in general weight was associated more strongly with the outcomes than length or height.

The associations with the conditional variables are shown in Table VII. Conditional length at age 2 years of age, a proxy for linear growth from birth to this age, was positively associated with IQ, years of schooling, and income at 30 years of age. The associations were stronger for linear growth than for relative weight at 2 years of age, and the latter association with income was not significant after adjustment. Neither conditional height nor weight at 4 years of age were associated with any of the outcomes in the adjusted analyses.

Table VIII shows the interactions between size at birth and the conditional measures. Only 1 of the 4 interactions was significant (*P* = .04). Conditional length at 4 years of age was associated positively with IQ at 30 years of age only among children whose birthweight for gestational age *z*-score was in the lowest tertile.

## Discussion

In this population-based prospective birth cohort, we observed that attained size (birthweight, weight for age and length, or height for age) are associated with IQ, years of schooling, and income at 30 years of age after controlling for several confounding variables. Associations tended to be stronger for attained weight than for length or height, and were present at both 2 and 4 years of age. In contrast, the picture emerging from conditional analyses is more refined. Linear growth was associated more strongly with the 3 outcomes than relative weight gain, for which there was no evidence of benefit after the age of 2 years of age. Associations with IQ and years of schooling tended to be stronger than those with income. Nevertheless, income was 20% higher in the group whose conditional length was ≥1 SD above what was expected, compared with those ≥1 SD below the expected. Weaker associations with income were expected because of the longer causal chain that presumably links early growth to adult earnings. We also sought interactions between size at birth and postnatal growth. Linear growth from 2 to 4 years of age was related to increased IQ among children with lower birthweights according to gestational age, suggesting potential benefits of late catch-up among those with intrauterine growth restriction, with positive consequences on human capital.

Our analyses report upon findings based on 2 types of anthropometric variables. Attained size (as weight for age or length/height for age) indicates cumulative growth or weight gain from conception to the age of measurement. Conditional analyses, in contrast, allow investigation of the effect of growth in different time periods. Conditional variables at 2 years of age indicate growth from birth to this age, and those at 4 years of age reflect changes from 2-4 years of age. Also, attained weight for age reflects both linear growth and relative weight gain above and beyond what would have been expected from linear growth alone. These distinctions explain why the 2 sets of analyses show apparently contradictory findings.

With respect to study limitations, subjects included in the analyses were more likely to be female and to belong to the intermediate family income groups when compared with the full cohort. The lower representation of children born with low birthweight and in the poorest income group is associated with their higher mortality in infancy. In contrast, birthweight and nutritional status in childhood were not associated with follow-up rate at the 2012-2013 visit.^29^ Furthermore, the regression coefficients for nutritional status in childhood was similar among those included and not included in the conditional growth analyses (*P* = .31). Therefore, selection bias is unlikely.

Residual confounding should be considered, because socioeconomic conditions are associated with the exposures and the outcomes.^35^, ^36^ When we controlled for several confounders measured soon after birth or in childhood in the analyses, which are positively associated with growth and with adult IQ and income,^37^ as expected, the regression coefficients were attenuated. The strongest argument against residual confounding is that the associations between conditional measures and the outcomes were specific—that is, they were higher for linear growth than for relative weight, and higher at 2 years of age than at 4 years of age. Residual confounding cannot account for such specificity in the results. Moreover, the observed association should not be considered as being owing to residual confounding by maternal intelligence and home stimulation. We controlled for several socioeconomic variables and breastfeeding, which are highly correlated with these unmeasured confounders.^37^ Therefore, a noncausal pathway has been closed.

Anthropometric assessments were carried out by trained interviewers and most of the confounding variables were measured in the perinatal study or in childhood, with a short recall. These measures, therefore, decreased measurement error and residual confounding. Furthermore, we used a standardized test to assess IQ.

Similar to our findings, most studies evaluating the association of growth at different moments in the life cycle with school performance or cognition reported positive associations with early growth, whereas late growth showed no or a weak association.^21^, ^22^, ^23^, ^24^, ^25^, ^26^ These findings are biologically plausible. Brain growth spurt occurs between the last trimester of pregnancy and at about 3-4 years of age, and growth velocity is higher in the first months of life.^38^ Indeed, most studies on the effect of birthweight and weight gain in childhood found that school achievement or IQ was associated more strongly with growth in childhood than intrauterine growth.^21^, ^22^, ^23^, ^24^, ^39^

This study on adult intelligence attempted to disentangle the effect of early linear growth from that of weight gain. Weight gain is a combination of linear growth and changes in soft tissue, and conditional relative weight gain represents the weight gain that is not owing to linear growth. We observed that linear growth in early childhood has positive long-term consequences on human capital, not only by improving IQ, but also increasing years of schooling and earning ability. In contrast, relative weight gain had a small impact on IQ and no effect on income. The regression coefficients for linear growth and relative weight gain at a given age may be compared, because both conditional growth variables are expressed as SD scores. In a pooled analysis of 5 cohorts from low- and middle-income countries (including data from the 23-year follow-up visit of the 1982 Pelotas cohort), Adair et al^9^ reported that birthweight, conditional length, and relative conditional weight at 2 years of age were associated inversely with the odds of failing to complete high school, and that these associations were strongest for conditional length gain. Our results are consistent with these earlier findings and, by showing a link with intelligence, the plausibility of the association with years of schooling is strengthened. As mentioned, brain growth velocity is higher in the first months of life,^38^ which would explain the specific association of early linear growth with IQ, years of schooling, and income.

Our results support the relevance of monitoring and promoting linear growth and shows that additional weight gain, after taking into account linear growth, has a small impact on human capital. Therefore, nutrition intervention programs should aim at promoting linear growth instead of weight gain, as well as intrauterine growth. And these interventions should focus in the first 1000 days of life, because they will have long-term consequences on human capital, by increasing IQ, years of schooling, and earning ability.

## Figures and Tables

**Table I:** Baseline characteristics of the cohort members according to inclusion in the conditional growth analyses

	Included in the conditional growth analyses				
	Yes		No		*P* value
	N	%	N	%	
Sex					
Male	1199	48.4	1839	53.5	<.001
Female	1278	51.6	1598	46.5	
Family income at birth(minimum wage)					<.001
≤1	414	16.8	874	25.6	
1.1-3	1222	49.4	1567	45.9	
3.1-6	532	21.5	559	16.4	
6.1-10	165	6.7	217	6.4	
>10	137	5.6	198	5.8	
Maternal years of schooling					<.001
0-4	704	28.4	1256	36.6	
5-8	1086	43.9	1368	39.9	
9-11	290	11.7	364	10.6	
≥12	395	16.0	444	12.9	
Birthweight (g)					<.001
<2500	147	5.9	387	11.3	
2500-2999	579	23.4	814	23.7	
3000-3499	948	38.3	1272	37.1	
≥3500	803	32.4	959	27.9	
Total*	2477		3437		

* For some variables, the number of subjects might not sum to the subjects included or not in the conditional growth analysis because of missing information.

**Table II:** Characteristics of study sample

	Mean (SD)	n	%
Variables measured at birth			
Monthly family income (minimum wage)			
≤1		706	19.6
1.1-3		1772	49.3
3.1-6		707	19.7
6.1-10		218	6.1
>10		191	5.3
Maternal education (y)			
0-4		1154	32.0
5-8		1557	43.2
9-11		394	10.9
≥12		501	13.9
Maternal skin color			
White		2969	82.2
Non-white		641	17.8
Birthweight (g)			
<2500		259	7.2
2500-2999		862	23.9
3000-3499		1356	37.5
≥3500		1133	31.4
Gestational age (wk)			
≤36		164	5.6
37-38		645	22.1
≥39		2106	72.3
Birthweight for gestational age (*z*-score)			
<−1.28 SD		413	14.2
−1.28-0 SD		1296	44.4
>0 SD		1205	41.4
Maternal smoking during pregnancy			
Yes		1262	35.0
No		2342	65.0
Variables measured in childhood			
Household assets index score (points)	0.34 (−0.55 to 0.68)*		
Paternal education (y)			
0-4		863	27.8
5-8		1415	45.6
9-11		405	13.1
≥12		419	13.5
Breastfeeding duration (mo)			
<1		736	21.1
1-2.9		895	25.6
3-5.9		808	23.1
≥6		1054	30.2
Height-for-age *z*-score at 2 years			
≤−2		424	12.8
−1.99 to −1		821	24.8
−0.99 to 1		1818	55.1
>1		242	7.3
Weight-for-age *z*-score at 2 years			
≤−2		88	2.7
−1.99 to −1		356	10.8
−0.99 to 1		2225	67.3
>1		637	19.3
Height-for-age *z*-score at 4 years			
≤−2		338	10.4
−1.99 to −1		842	26.0
−0.99 to 1		1842	56.9
>1		216	6.7
Weight-for-age *z*-score at 4 years			
≤−2		65	2.0
−1.99 to −1		431	13.3
−0.99 to 1		2252	69.6
>1		488	15.1
Variables measured at 30 years			
IQ	98.0 (12.6)		
Educational attainment (y)	11.4 (4.1)		
Monthly income (R$)	1000 (530-1890)*		

* Median and interquartile range.

**Table III:** Prevalence of birth conditions, nutritional status in childhood and rapid growth in childhood according to socioeconomic, demographic, and maternal smoking variables

	Low birthweight (%)	Preterm birth (%)	Small-for-gestational age (%)	Weight-for-age *z*-score at 2 years ≤−2 (%)	Height-for-age *z*-score at 2 years ≤−2 (%)	Weight-for-age *z*-score at 4 years ≤−2 (%)	Height-for-age *z*-score at 4 years ≤−2 (%)	Conditional length *z*-score at 2 years—≥1 (%)	Conditional weight *z*-score at 2 years—≥1 (%)	Conditional height *z*-score at 4 years—≥1 (%)	Conditional weight *z*-score at 4 years—≥1 (%)
Family income at birth(in minimum wages)	*P* < .001	*P* = .9	*P* < .00	*P* < .001	*P* < .001	*P* < .00	*P* < .00	*P* < .001	*P* = .57	*P* = .42	*P* = .21
≤1	11.8	56.1	118.8	6.6	23.9	14.8	122.0	5.6	16.2	14.3	16.0
1.1-3	6.2	5.4	15.4	2.5	13.2	1.8	9.5	13.1	13.9	14.0	12.5
3.1-6	6.1	5.9	10.9	0.5	6.2	0.5	5.3	22.4	16.8	16.6	15.8
6.1-10	6.0	6.2	8.3	0.5	4.6	1.0	5.7	23.2	15.9	14.6	13.4
≥10	4.7	4.8	9.0	0.6	2.9	0.6	1.8	27.0	19.7	19.0	13.1
Maternal years of schooling at birth	*P* = .03	*P* = .7	*P* = .00	*P* < .001	*P* < .001	*P* < .00	*P* < .00	*P* < .001	*P* = .48	*P* = .17	*P* = .43
0-4	7.8	55.3	515.5	5.1	20.7	14.6	117.0	9.8	15.8	12.4	14.5
5-8	8.0	6.1	15.7	2.3	12.3	1.0	9.6	13.3	14.9	15.7	14.7
9-11	4.3	4.7	11.0	0.3	3.6	0.6	4.0	20.1	15.2	16.3	11.8
≥12	5.6	5.6	10.0	0.2	3.7	0.4	3.1	26.6	16.0	16.2	12.7
Maternal skin color	*P* = .06	*P* = .7	*P* = .61	*P* < .001	*P* < .001	*P* < .00	*P* < .00	*P* < .001	*P* = .22	*P* = .18	*P* = .88
White	6.8	05.7	14.0	2.2	11.0	11.9	19.2	16.2	15.4	14.5	13.8
Non-white	8.9	5.3	14.9	4.8	21.5	2.4	16.0	10.4	15.1	17.1	14.9
Household assets index (quintiles)	*P* = .02	*P* = .3	*P* < .00	*P* < .001	*P* < .001	*P* < .00	*P* < .00	*P* < .001	*P* = .41	*P* = .36	*P* = .22
First	10.0	16.7	118.9	6.6	25.7	14.4	122.6	6.0	14.6	12.2	13.7
Second	7.9	5.4	17.6	2.8	17.0	3.2	11.9	11.2	14.4	14.4	12.6
Third	6.6	5.5	13.3	1.7	8.2	1.2	7.5	15.0	16.7	15.2	12.5
Fourth	4.1	3.8	10.5	1.3	7.0	0.7	4.7	23.0	17.5	17.1	16.7
Fifth	7.0	7.3	9.4	1.0	6.0	0.8	4.7	25.3	13.1	16.6	17.5
Maternal smoking in the pregnancy	*P* < .001	*P* = .6	*P* < .00	*P* < .001	*P* < .001	*P* < .00	*P* < .00	*P* = .01	*P* = .004	*P* = .75	*P* = .91
No	5.8	75.5	111.1	2.6	10.5	11.9	18.8	16.0	14.4	15.1	14.0
Yes	9.8	5.9	20.4	2.8	17.1	2.3	13.5	13.8	17.5	14.6	13.9

**Table IV:** IQ, years of schooling, and income at 30 years of age, according to socioeconomic, demographic, and maternal smoking variables in the pregnancy

	IQ (points), mean (95% CI)	Years of schooling, mean (95% CI)	Monthly income (R$), mean (95% CI)
Family income at birth (minimum wage)	*P* < .001	*P* < .001	*P* < .001
≤1	91.5(90.7-92.4)	8.9(8.6-9.1)	940(855-1024)
1.1-3	96.6(96.0-97.1)	10.7(10.6-10.9)	1255(1191-1320)
3.1-6	102.0(101.2-102.9)	13.1(12.8-13.4)	1894(1750-2038)
6.1-10	106.7(105.2-108.2)	14.5(14.1-14.9)	2583(2260-2907)
≥10	110.4(108.9-112.0)	15.8(15.3-16.2)	3208(2787-3628)
Maternal years of schooling at birth	*P* < .001	*P* < .001	*P* < .001
0-4	92.2(91.5-92.9)	9.2(9.0-9.4)	997(931-1062)
5-8	97.5(97.0-98.1)	11.1(10.9-11.3)	1356(1281-1430)
9-11	103.2(102.1-104.3)	13.2(12.9-13.5)	1870(1679-2063)
≥12	108.6(107.7-109.5)	15.3(15.1-15.6)	2846(2614-3078)
Maternal skin color	*P* < .001	*P* < .001	*P* < .001
White	99.2(98.8-99.7)	11.6(11.5-11.8)	1616(1548-1683)
Non-white	92.1(91.2-93.0)	9.9(9.6-10.2)	977(906-1048)
Household assets index(quintiles)	*P* < .001	*P* < .001	*P* < .001
First	90.0(89.1-91.0)	8.4(8.1-8.7)	868(797-938)
Second	95.2(94.3-96.0)	10.3(10.0-10.6)	1219(1117-1320)
Third	100.4(99.7-101.0)	12.1(11.9-12.4)	1564(1466-1662)
Fourth	101.3(100.0-102.6)	12.7(12.3-13.1)	1762(1556-1967)
Fifth	104.2(103.1-105.2)	13.7(13.3-14.0)	2321(2100-2542)
Maternal smoking during pregnancy	*P* < .001	*P* < .001	*P* = .001
No	99.1(98.6-99.6)	11.7(11.5-11.8)	1573(1499-1648)
Yes	95.9(95.2-96.6)	10.7(10.5-10.9)	1371(1282-1460)

**Table V:** IQ, years of schooling, and income at 30 years, according to birth conditions

	IQ (points)		Years of schooling		Monthly income (R$)	
	Mean (95% CI)	Adjusted regression coefficient (95% CI)	Mean (95% CI)	Adjusted regression coefficient (95% CI)	Mean (95% CI)	Adjusted regression coefficient (95% CI)
Birthweight (g)	*P* < .001*	*P* < .001*	*P* < .001*	*P* = .003*	*P* < .001*	*P* < .001*
<2500	94.5(92.8to96.1)	Reference(0)	10.5(10.0to11.0)	Reference(0)	1190(1009to1370)	Reference(0)
2500-2999	95.7(94.8to96.5)	1.02(−0.47to2.51)	10.6(10.4to10.9)	0.04(−0.43to0.52)	1266(1161to1370)	69(−159to296)
3000-3499	98.5(97.8to99.1)	2.22(0.79to3.65)	11.4(11.2to11.7)	0.29(−0.17to0.75)	1538(1444to1632)	193(−26to412)
≥3500	99.9(99.2to100.6)	2.93(1.47to4.39)	11.9(11.6to12.1)	0.48(0.01to0.95)	1709(1597to1821)	315(91to538)
Preterm birth	*P* = .29	*P* = .29	*P* = .21	*P* = .18	*P* = .84	*P* = .85
No	98.8(98.4to99.3)	Reference(0)	11.6(11.5to11.8)	Reference(0)	1592(1523to1660)	Reference(0)
Yes	97.8(95.8to99.8)	−0.86(−2.56to0.84)	11.2(10.6to11.8)	−0.36(−0.90to0.17)	1562(1263to1861)	−12(−283to259)
Birthweight for gestational age(*z*-score)	*P* < .001*	*P* = .001*	*P* < .001*	*P* < .001*	*P* < .001*	*P* = .10*
<−1.28	95.7(94.5to96.9)	Reference(0)	10.5(10.2to10.9)	Reference(0)	1311(1159to1464)	Reference(0)
−1.28-0	98.1(97.4to98.8)	1.26(0.06to2.46)	11.3(11.1to11.5)	0.35(−0.02to0.72)	1558(1461to1655)	138(−53to328)
>0	100.6(99.9to101.3)	2.33(1.10to3.55)	12.3(12.0to12.5)	0.82(0.44to1.21)	1721(1611to1831)	179(−16to374)

Adjusted for family income at birth, parental years of schooling, household asset index score, maternal skin color, and maternal smoking during pregnancy.

* *P*-value for linear trend.

**Table VI:** IQ, years of schooling, and income at 30 years, according to nutritional status in childhood

	IQ (points)		Years of schooling		Monthly income (R$)	
	Mean (95% CI)	Adjusted regression coefficient (95% CI)	Mean (95% CI)	Adjusted regression coefficient (95% CI)	Mean (95% CI)	Adjusted regression coefficient (95% CI)
Weight-for-age *z*-score at 2 years	*P* < .001*	*P* < .001*	*P* < .001*	*P* < .001*	*P* < .001*	*P* = .003*
≤−2	88.1(85.7to90.5)	Reference(0)	7.6(6.8to8.4)	Reference(0)	624(500to748)	Reference(0)
−1.99to−1	92.3(91.1to93.6)	2.33(−0.14to4.80)	9.5(9.1to9.9)	1.28(0.51to2.05)	1055(925to1185)	226(−147to599)
−0.99to1	98.0(97.5to98.6)	4.29(1.99to6.59)	11.3(11.2to11.5)	1.92(1.20to2.64)	1482(1410to1554)	258(−89to605)
>1	102.8(101.9to103.7)	5.70(3.21to8.18)	13.0(12.7to13.3)	2.50(1.72to3.27)	1929(1767to2090)	349(−26to724)
Height-for-age *z*-score at 2 years	*P* < .001*	*P* < .001*	*P* < .001*	*P* < .001*	*P* < .001*	*P* = .67†
≤−2	90.6(89.4to91.8)	Reference(0)	8.8(8.4to9.2)	Reference(0)	1022(900to1143)	Reference(0)
−1.99to−1	95.4(94.6to96.3)	2.22(0.96to3.48)	10.4(10.1to10.7)	0.80(0.40to1.20)	1258(1158to1357)	−41(−235to153)
−0.99to1	100.0(99.5to100.6)	3.83(2.62to5.05)	12.0(11.8to12.2)	1.43(1.04to1.81)	1624(1537to1710)	−14(−202to173)
>1	105.2(103.9to106.5)	4.77(2.93to6.62)	13.8(13.4to14.2)	1.80(1.22to2.38)	2208(1928to2489)	112(−173to397)
Weight-for-age *z*-score at 4 years	*P* < .001*	*P* < .001*	*P* < .001*	*P* < .001*	*P* < .001*	*P* = .005*
≤−2	86.1(83.2to89.0)	Reference(0)	7.7(6.8to8.6)	Reference(0)	697(526to868)	Reference(0)
−1.99to−1	93.3(92.1to94.4)	4.53(1.70to7.36)	9.8(9.5to10.2)	1.33(0.44to2.21)	977(867to1087)	9(−407to425)
−0.99to1	98.0(97.5to98.5)	5.91(3.20to8.63)	11.3(11.2to11.5)	1.68(0.83to2.53)	1506(1435to1578)	213(−186to613)
>1	103.6(102.6to104.6)	7.97(5.07to10.87)	13.3(12.9to13.6)	2.43(1.52to3.34)	1968(1783to2153)	328(−100to757)
Height-for-age *z*-score at 4 years	*P* < .001*	*P* < .001*	*P* < .001*	*P* < .001*	*P* < .001*	*P* = .04*
≤−2	90.7(89.3to92.0)	Reference(0)	8.8(8.4to9.3)	Reference(0)	866(756to975)	Reference(0)
−1.99to−1	94.8(94.0to95.7)	1.85(0.45to3.25)	10.2(9.9to10.5)	0.67(0.23to1.10)	1259(1158to1362)	152(−53to358)
−0.99to1	100.0(99.4to100.5)	3.71(2.35to5.07)	12.1(11.9to12.3)	1.33(0.91to1.76)	1638(1553to1722)	192(−7to391)
>1	105.0(103.6to106.4)	5.33(3.32to7.33)	13.7(13.2to14.1)	1.74(1.12to2.37)	2122(1839to2405)	316(19to613)

Adjusted for family income at birth, parental years of schooling, household assets index, maternal skin color, maternal smoking during pregnancy, breastfeeding duration, and birthweight.

* *P* value for linear trend.

† *P* value for heterogeneity.

**Table VII:** IQ, years of schooling, and income at 30 years, according to conditional growth in childhood

	Regression coefficient (95% CI)					
	IQ (points)		Years of schooling		Monthly income (R$)	
	Crude	Adjusted	Crude	Adjusted	Crude	Adjusted
Conditional lengthat 2 years (*z*-score)	*P* < .001*	*P* < .001*	*P* < .001*	*P* < .001*	*P* < .001*	*P* = .06†
≤−1	Reference(0)	Reference(0)	Reference(0)	Reference(0)	Reference(0)	Reference(0)
−0.99to−0.01	4.36(2.90to5.83)	2.11(0.79to3.42)	1.50(1.03to1.97)	0.73(0.32to1.13)	336(115to558)	106(−105to317)
0.00to0.99	7.95(6.48to9.41)	3.98(2.64to5.32)	2.68(2.22to3.15)	1.30(0.89to1.72)	458(236to680)	33(−183to249)
≥1	10.52(8.79to12.24)	4.41(2.79to6.02)	3.80(3.25to4.35)	1.62(1.12to2.12)	961(700to1221)	299(39to558)
Conditional relative weightat 2 years(*z*-score)	*P* = .02*	*P* = .04*	*P* = .004†	*P* = .04†	*P* = .02*	*P* = .05*
≤−1	Reference(0)	Reference(0)	Reference(0)	Reference(0)	Reference(0)	Reference(0)
−0.99to−0.01	1.08(−0.37to2.53)	0.26(−1.03to1.55)	0.57(0.11to1.03)	0.26(−0.14to0.66)	61(−159to280)	−38(−245to170)
0.00to0.99	1.30(−0.17to2.77)	0.60(−0.71to1.91)	0.84(0.37to1.30)	0.54(0.14to0.95)	184(−39to407)	91(−120to301)
≥1	2.09(0.38to3.81)	1.49(−0.04to3.01)	0.78(0.24to1.33)	0.50(0.03to0.97)	258(−1to518)	170(−76to416)
Conditional heightat 4 years(*z*-score)	*P* < .001†	*P* = .51†	*P* < .001†	*P* = .56†	*P* < .001†	*P* = .15†
≤−1	Reference(0)	Reference(0)	Reference(0)	Reference(0)	Reference(0)	Reference(0)
−0.99to−0.01	2.05(0.58to3.52)	0.63(−0.68to1.95)	0.68(0.22to1.15)	0.14(−0.27to0.54)	367(145to589)	214(3to425)
0.00to0.99	3.03(1.55to4.51)	1.03(−0.31to2.36)	0.98(0.51to1.45)	0.22(−0.19to0.63)	461(237to686)	243(29to457)
≥1	2.50(0.76to4.24)	0.74(−0.83to2.31)	0.71(0.15to1.26)	−0.03(−0.51to0.45)	376(112to639)	179(−73to431)
Conditional relative weight4 years(*z*-score)	*P* = .07*	*P* = .15†	*P* = .09*	*P* = .24*	*P* = .15†	*P* = .17†
≤−1	Reference(0)	Reference(0)	Reference(0)	Reference(0)	Reference(0)	Reference(0)
−0.99to−0.01	−0.30(−1.81to1.20)	0.05(−1.30to1.39)	−0.24(−0.72to0.24)	−0.10(−0.51to0.32)	−15(−243to214)	34(−182to250)
0.00to0.99	−0.14(−1.69to1.41)	0.17(−1.21to1.55)	−0.24(−0.74to0.25)	−0.11(−0.54to0.31)	151(−84to386)	187(−35to409)
≥1	−1.99(−3.80to−0.17)	−1.33(−2.95to0.29)	−0.57(−1.15to0.01)	−0.32(−0.82to0.18)	−65(−341to210)	23(−238to284)

Adjusted for family income at birth, parental years of schooling, household index score, maternal skin color, maternal smoking during pregnancy, birthweight and breastfeeding duration.

* Test for linear trend.

† Test for heterogeneity.

**Table VIII:** Conditional growth analyses of IQ at 30 years according to birthweight for gestational age *z*-score*

	Adjusted regression coefficient (95% CI)			*P*-value for interaction
	Birthweight for gestational age *z*-score			
	First tertile	Second tertile	Third tertile	
Conditional length 2 years	1.51(0.74to2.28)	1.02(0.24to1.80)	2.35(1.54to3.15)	.44
Conditional relative weight 2 years	0.26(−0.45to0.98)	0.33(−0.42to1.07)	0.88(0.15to1.62)	.20
Conditional height 4 years	0.98(0.25to1.71)	0.31(−0.45to1.07)	−0.24(−0.97to0.50)	.04
Conditional relative weight 4 years	−0.34(−1.08to0.39)	0.00(−0.72to0.73)	−0.86(−1.58to−0.14)	.30

Adjusted for family income at birth, parental years of schooling, household index score, maternal skin color, maternal smoking during pregnancy, and breastfeeding duration.

* Results are expressed as change in IQ(points) associated with one *z*-score of the conditional variable.
